# Use of neuroendoscopy in metastatic brain tumours: A systematic review

**DOI:** 10.3892/mi.2026.315

**Published:** 2026-04-14

**Authors:** Basem Fouda, Matthew Laffey, Usama Al-Abri, Yuriy Dovhan, Tariq Al-Saadi

**Affiliations:** 1 School of Medicine, Trinity College Dublin, The University of Dublin, Dublin, D02 PN40, Ireland; 2 Department of Neurosurgery - Cedars-Sinai Medical Centre, Los Angeles, CA 90048, USA

**Keywords:** neuroendoscopy, metastatic brain tumours, brain metastases, minimally invasive neurosurgery, surgical outcomes, intracranial metastasis

## Abstract

Neuroendoscopy allows the performance of biopsy and the resection of metastatic brain lesions using a minimally invasive approach. The present systematic review aimed to survey the existing evidence surrounding the use of neuroendoscopic techniques in the setting of metastatic brain lesions. A systematic search of the available literature was performed using the approach outlined by the Preferred Reporting Items for Systematic Reviews and Meta-Analyses (PRISMA) method. The search identified 34 studies that enrolled 150 participants. The average age of the patients was 57 years (range, 37-80 years), and the female-to-male ratio was 51 to 54. The most common primary sites were the lung (42/113), breast (18/113) and melanoma (16/113). Supratentorial tumours represented 93 of the 115 cases. Of the 34 included studies, the goal of the procedure was resection in 23 studies, biopsy in six studies (with ventriculostomy in 3), and both in five studies. The degree of resection was reported for 93 participants, in which a gross, near and subtotal resection was achieved in 60, 13 and 20 cases, respectively. Overall, 13 studies encompassing 34 participants reported that 28 (82%) patients demonstrated post-operative symptomatic improvement following resection. The overall survival was reported for 50 patients following resection across four studies, yielding a weighted average of 12.8 months. To the best of our knowledge, the present study represents the first comprehensive systematic assessment of the existing evidence surrounding the role of neuroendoscopy for brain metastases. The findings presented herein demonstrate promise for the role of neuroendoscopy in this setting; however, the quality and quantity of evidence limit the drawing of strong conclusions.

## Introduction

Brain metastasis is considered a major complication of the later stages of cancer. This is associated with various signs and symptoms, including headaches, seizures, weakness in the arms or legs, loss of balance, memory loss and speech disruptions (1). The management of these metastatic lesions has always been a challenge. There are different approaches employed in treating metastatic brain tumours, including the following:

Radiation therapy uses X-rays, protons and other high-energy electron beams to eliminate cancer cells. The two most known techniques are stereotactic radiosurgery (SRS) and whole-brain radiation therapy (WBRT). SRS provides precise radiation towards the cancerous cells and is performed in a few sessions, whereas the radiation in WBRT is applied to the whole brain and requires 10-15 sessions (2). Traditionally, SRS was limited to patients with up to four brain metastases. However, it is increasingly being considered a viable treatment option for those with more numerous metastases, with a recent study demonstrating a promising safety and efficacy profile in patients with ≥15 brain metastases (3).

Chemotherapy is limited as the majority of chemotherapeutic agents cannot cross the blood-brain barrier. However, it is still considered a viable option. Extensive research has been performed in an aim to improve drug delivery to the brain (2).

Targeted therapy is one of the newest methods in the treatment of cancer. The agents used in this type of therapy can cross the blood-brain barrier and can identify cancerous cells with minimal harm to normal cells. It is usually co-administered with radiation therapy or administered following surgery to eliminate the remaining cancerous cells (1).

Recently, neuroendoscopy has gained increasing attention in the field of neurosurgery, being used for the treatment of a wide range of conditions including hydrocephalus, intracranial cysts, craniosynostosis, intraventricular tumours, and skull base tumours (4,5). Due to its minimally invasive technique, while maintaining effectiveness and comparative safety, it has become the preferred surgical approach for the management of many intracranial diseases (5). A recent single-centre retrospective study surveying 318 neuroendoscopic procedures for intracranial pathologies reported that only 5.4% of cases were affected by an early surgical adverse event while 3.1% were affected by a non-surgical adverse event (5).

The present systematic review aimed to summarise the available literature surrounding neuroendoscopic procedures in patients diagnosed with metastatic brain tumours. In particular, the authors were interested in summarising the available evidence regarding the characteristics of patients selected for this technique, technical details regarding the procedure in this patient population, and evaluating the evidence regarding the outcomes of these neuroendoscopic procedures.

## Data and methods

The present systematic review was undertaken using the approach outlined by the Preferred Reporting Items for Systematic Reviews and Meta-Analyses (PRISMA) method (6). Articles assessed as part of the present systematic review were identified from a search of the PubMed library electronic database performed on June 1, 2022. The search string utilised was: ‘(Endoscop*[Title/Abstract] OR Neuroendoscop*[Title/Abstract]) AND (Metast*[Title/Abstract]) AND (Brain[Title/Abstract])’. Asterisks (‘*’) and Boolean operators ‘AND’ and ‘OR’ are established tools used to search the PubMed library and were used to enhance search yield.

Studies were included or excluded from analysis in the present systematic review in accordance with predefined inclusion and exclusion criteria. Following a review of the existing literature and discussions with an expert in this field, the present systematic review decided to focus its search on the use of endoscopic techniques in the management of metastatic brain tumours.

The inclusion criteria were the following: i) Studies involving only adult (≥18 years) participants; ii) studies in which an endoscopic procedure was conducted on a metastatic brain lesion; iii) studies that reported specific demographic, procedural, or outcome parameters for those with metastatic brain lesions who received endoscopic intervention(s).

The exclusion criteria were as follows: i) Studies involving non-human participants; ii) studies not available in the English language; iii) studies that did not report demographic, procedural, or outcome parameters for those with metastatic brain lesions who received an endoscopic intervention. Duplicate studies were removed, and for instances in which multiple papers were written on the same trial, the study with the largest sample size was selected.

Data recorded and discussed as part of the present systematic review arose from distinct article selection and data extraction steps. All articles were assessed against the previously described inclusion and exclusion criteria at both title and abstract and full-text screening stages by two independent reviewers. Conflicts were resolved by a third-party reviewer. Following study selection, the following data were extracted from the included studies: i) Citation data; ii) study design characteristics; iii) study population characteristics (number, disease, intervention, age, sex, presenting symptoms, primary cancer histology, lesion location and lesion size); iv) intervention characteristics (aim/indication, preoperative assessment, operative route, operative technique, adjunctive tools and therapies, post-operative assessment; and v) outcome measures (procedure success, duration, adverse events, length of hospital stay (LOS), mean follow-up, and clinical outcomes). Details surrounding the number of articles screened at each stage and the extracted data are presented in the Results section.

## Results

### Study selection

**Figure 1:**
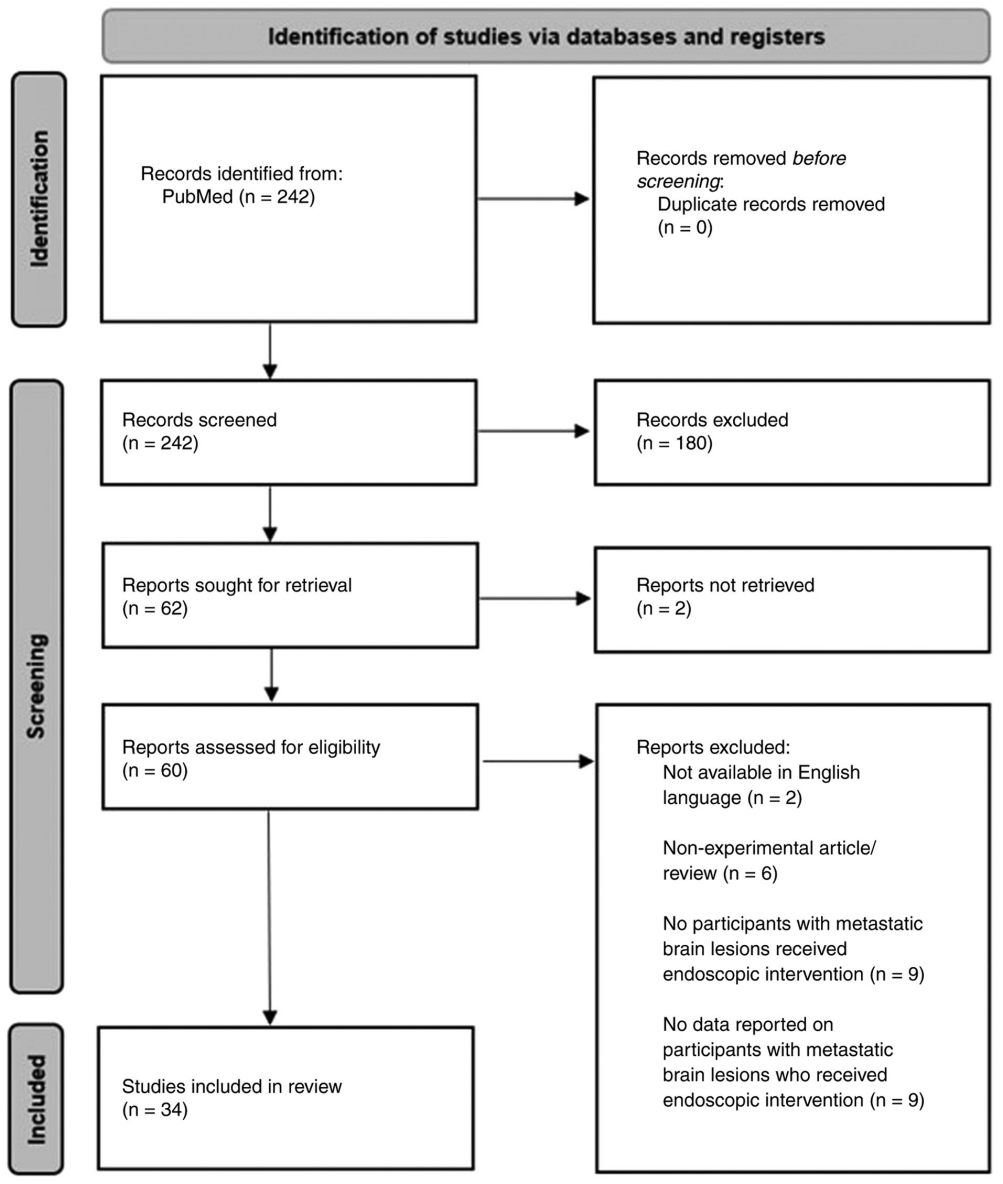
PRISMA diagram detailing identification and screening processes used to select studies. Format based on that outlined in the most recent PRISMA statement provided by Page *et al* (6).

Findings from the electronic database search and article screening stages are outlined in accordance with the most recent PRISMA guidelines published by Page *et al* (6) in Fig. 1. The literature search identified 242 articles. Covidence software identified none of these as duplicates. Title and abstract screening identified 62 articles meeting the inclusion and exclusion criteria for full-text review. Upon full-text review, studies that could not be retrieved, duplicate studies, and those that did not meet the inclusion and exclusion criteria were identified and removed, as illustrated in Fig. 1. Ultimately, 34 studies were selected for data extraction and subsequent analysis as part of the present systematic review. These are listed in Table I (7-40).

### Patient demographics

Demographic data for the included studies are outlined in Table SI. In total, the included studies reported data on 686 participants, 150 of whom received an endoscopic intervention on a non-primary brain tumour. Within this cohort, the average age of the patients was 57 years (range, 37-80 years), and the female-to-male ratio was 51 to 54. Patient presenting symptoms were reported in 18 studies and are outlined below. Tumour histology was reported by 27 of the included 34 studies for a total of 113 patients. Metastases from the lung (42/113), breast (18/113) and melanoma (16/113) represented the most common diagnoses. The remaining tumours were either unclassified adenocarcinomas (10/113) or small numbers of other tumour types, including renal (5/113), GIT (5/113) and bladder (4/113).

Lesion location was reported in 29 studies for a total of 115 patients and was subclassified with relation to the tentorium cerebelli for the purposes of the present systematic review. Supratentorial tumours represented 93 of the 115 cases and primarily comprised tumours located in the cortex (38/115), sella (14/115), and ventricles (10/115). Alternative locations included subcortical (9/115), sinuses (4/115) and other/not otherwise specified (18/115). Infratentorial tumours comprised 22 of the 115 described lesions and primarily comprised those located in the cerebellum (10/115). Lesion size was reported in 13 of the included 34 studies. Maximum dimension was the most commonly reported metric, with measurements ranging from 10 to 41 mm across 8 studies.

### Procedures performed

Details on the operative procedure undertaken are summarised in Table SII. Of the 34 included studies, the goal of the procedure was resection in 23, biopsy in six studies (with ventriculostomy in 3), and both in 5 studies. The procedure was undertaken using a fully endoscopic approach in 26 studies and in combination with microsurgery in eight studies. The majority of procedures were undertaken using a transcranial route via a corticotomy (20/32). Alternative routes included extended endonasal (8/32), transorbital (3/32) and a combined transorbital and extended endonasal approach utilised in one study. Procedures were undertaken using rigid endoscopes in 18 of the 21 studies that reported this. Furthermore, intraoperative fluorescence was utilized in procedures undertaken in 7 of the included studies.

Table SII also includes a summary of the pre- and post-operative assessments undertaken alongside the use of adjuvant therapy. Magnetic Resonance Imaging (MRI) was utilised in pre-operative assessment in all 32 studies that reported on this. Computed Tomography (CT) imaging was also performed in the preoperative assessment, utilised in nine studies. Similarly, MRI and CT scan were used in the post-operative assessment of patients in 26 and 11 of the 27 studies reporting this variable, respectively. The use of adjunctive chemotherapy and radiotherapy was poorly reported (10 and 13 studies, respectively), with 5 and 11 studies reporting their use, respectively.

### Outcomes of procedures

Periprocedural outcomes reported by the studies included in this analysis are presented in Table SIII. Procedural time was reported in two studies, yielding an average of 122 min (range, 83-160 min). Procedural success was measured in terms of the extent of resection [gross total resection (GTR), near total resection (NTR) and subtotal resection (STR)] for resections and obtaining sufficient tissue for histological assessment for biopsies. Of the 28 studies reporting resection as a goal, 23 studies recorded the degree of resection in terms of the definitions of GTR, NTR and STR utilised by the present systematic review for a cohort of 93 patients. Within this cohort, GTR was achieved in 60 patients, NTR was achieved in 13, and STR was achieved in 20 patients. Furthermore, of the six studies that performed biopsies alone, the procedure was successful in all 7 patients.

Overall, 13 studies encompassing 34 patients reported that 28 (82%) patients demonstrated post-operative symptomatic improvement. The overall survival was reported for 50 patients across four studies, yielding a weighted average of 12.8 months.

Adverse events were classed as intraoperative and post-operative, and LOS was reported in days. Overall, a total of 22 participants experienced adverse events from a total of 124 participants across 15 studies. Intraoperatively, there were seven CSF leaks and one bleed requiring 400 ml transfusion, resulting in a total of eight intraoperative complications. Post-operatively, there was a total of 14 individuals who experienced post-operative adverse events, which included 3 cases of wound infection; 3 cases of panhypopituitarism, 2 cases of transient arm paresis; one post-operative CSF leak; one patient who suffered both a brain abscess and pulmonary embolism; one instance of re-cannulation where a second cannulation was required to adequately access the target lesion; one proximal optic radiation infarct; and two deaths in the 30 days following the procedure arising from multiorgan failure and pulmonary embolism, respectively. Post-operative LOS was reported for the metastatic cohort in one study in which the patient remained in hospital for 14 days after his surgery, before being discharged with outpatient follow-up.

## Discussion

Neuroendoscopy represents a promising avenue to facilitate the diagnosis and treatment of metastatic brain lesions both in isolation and in conjunction with microsurgical techniques. The present systematic review aimed to assess and summarise the existing evidence surrounding the role of neuroendoscopy in this setting. In undertaking the present systematic review, the authors assessed the existing evidence and have provided aggregate measures of the demographic factors of the study population, details of the operative procedures undertaken, and relevant outcome measures, including procedural success and complication rates. Throughout this section, the relevance of the findings concerning the existing literature in this space and the strengths and limitations of the study are discussed, and these are applied to yield recommendations for future research.

The initial literature search yielded 242 articles. Following the abstract and full-text screening, 34 studies were included for data extraction and subsequent analysis; these are listed in Table I. Across the 34 studies, there were 686 patients, of whom 150 patients had neuroendoscopic procedures performed for the resection or biopsy of metastatic brain tumours.

The number of patients relevant to the present systematic review in each study was low, with an average of 4.41 patients per study. The largest number of patients in one study was 26, while 15 of the included papers only had 1 patient who had undergone neuroendoscopy for the biopsy or resection of a metastatic brain tumour.

The extraction sheets were designed to contain the most critical characteristics and outcomes that the authors were interested in summarising. However, surprisingly, a number of the studies did not provide information on these outcomes that is specific to metastatic patients who had undergone neuroendoscopy (8,11-14,16,20-25,28,29,31-33,35). Most strikingly, only two studies, conducted by the same group, reported the total duration of surgery (24,25), while only one study reported LOS (7). Furthermore, data were limited for the total length of follow-up and detailed reports of clinical outcomes post-procedure. Likewise, the number of studies that reported on the lesion size, the use of fluorescence, adjuvant chemotherapy, or adjuvant radiotherapy was limited.

The average age of patients with metastatic brain disease who had undergone neuroendoscopy was 57.46 years (range, 37 to 80 years). This is consistent with data available demonstrating that the most common age for the incidence of brain metastases is the 6th decade of life (41). Furthermore, this is similar to that reported by other groups, such as Winther *et al* (42) who reported a median age of 63 years (range, 18-89 years) in their retrospective assessment of 373 patients who underwent conventional craniotomy and resection of single brain metastases.

A total of 28 of the 34 included studies utilised neuroendoscopy for the resection of tumours, while the remaining percentage only carried out biopsies. The procedural time in this series was reported in two studies, yielding an average of 122 min (range, 83-160 min). This is comparable to that reported in the study by Gupta *et al* (43); they reported a median operative time of 139 min (interquartile range, 98-193 min) following their assessment of 3,500 cases of craniotomy for resection of brain metastasis.

GTR was achieved in 64.51% of the cases, whereas in 13.98 and 21.50% of cases, NTR and STR were achieved, respectively. A recent retrospective study by Winther *et al* (42) examining patients with brain metastases, reported improved survival with GTR compared to STR, highlighting the importance of the extent of resection. The extent of resection rates reported in the present analysis is similar to those reported in the study by Winther *et al* (42), where they found that out of 373 patients, 64% achieved GTR, and 36% achieved STR. This indicates that with neuroendoscopic procedures, it is possible to achieve similar extents of resection to other techniques (42).

As regards clinical outcomes, it was found that 82.35% of patients were reported to have at least some level of symptomatic improvement. There was overall very poor reporting of overall survival for the included patients with metastatic brain tumours. The average of overall survival reported in four studies (50 patients) was 14.99 months (10,18,26,38). When weighted by the number of patients in each study, the average overall survival is 12.82 months. This is again in line with the study by Winther *et al* (42), which reported that the median overall survival was 11.0 months.

Adverse events were poorly reported in this series, with only 15 of the 34 included studies reporting adverse event outcomes on a subpopulation of 124 participants (8-10,15,17,21,24-26,31,32,37-40). Overall, a total of 22 patients suffered adverse events across intra-operative and post-operative time periods. Intra-operatively, there was one major haemorrhage requiring transfusion (0.8%) reported in this series, which is infrequent compared to that of 3.65% reported by Aziz *et al* (44) following their assessment of 40,883 patients who underwent craniotomy for brain tumour resection. There were seven intra-operative CSF leaks, however, only 1 participant (0.8%) went on to experience a post-operative CSF leak which is similar to that of 1% reported by Winther *et al* (42). With regards to post operative adverse events, the aggregate figure of 11.3% observed in the present study cohort is comparable to the 7% reported by Winther *et al* (42). Notably, 2 patients in the series examined in the present study died within 30 days of surgery (1.6%) due to pulmonary embolism and multiorgan failure respectively, which is less than the 3.7% of those who died in the 6 weeks following intervention in the series published by Winther *et al* (42)..

Neuroendoscopy provides several unique advantages compared to conventional microsurgical approaches, particularly in the setting of minimally invasive surgery and applications for label-free imaging techniques.

Minimally invasive surgery aims to limit unnecessary damage to intracranial and extracranial tissues by minimising the size of the access site, surgical corridor, and the degree of intraoperative tissue retraction utilised in neurosurgical procedures (45). In pursuit of this aim, neuroendoscopy demonstrates several clear advantages over microscopic and unassisted approaches through allowing a wide field of view, slim and flexible instrumentation, and improved illumination, which allow the use of longer, narrower, and tortuous surgical routes (45). While the use of minimally invasive surgical techniques requires careful patient selection and a significant learning curve, these approaches demonstrate clear advantages over open transcranial techniques in terms of decreased procedural morbidity and complication rate, quicker recovery time and improved cosmesis (45).

Furthermore, the use of neuro-endoscopes capable of label-free imaging techniques such as narrow band imaging (NBI) may allow intra-operative histopathological diagnosis and improve resection margins. NBI describes the use of optical filters that transmit only light of 540 nm (green) and 415 nm (blue) wavelengths, which are absorbed by haemoglobin, which provides coloration to tissues based on capillary density and architecture (46). Endoscopic narrow band imaging has demonstrated utility in the detection of a wide range of early cancers, including cancers of the head and neck, upper and lower gastrointestinal tract, urological and gynaecological tracts, and lungs and bronchus (46). NBI has also demonstrated applications in the setting of cerebral malignancy, such as that reported by Sasagawa *et al* (47), who found that neuroendoscopic NBI allowed for the intraoperative identification and biopsy of tumour surfaces which were not perceptible on white light imaging. Additionally, the development of endoscopes capable of other label-free imaging techniques, such as confocal laser endomicroscopy, coherent anti-stokes Raman scattering, two-photon excitation fluorescence and second harmonic generation also represents promising avenues to allow intraoperative diagnosis and improve tumour resection margins (48-51).

The present study is subject to several limitations. First, the majority of studies included in the present systematic review were retrospective in nature and involved small numbers of participants that meet the inclusion criteria. These factors should be taken into account when assessing the aggregate data and limit the strength by which recommendations can be made based on this evidence. Second, the present systematic review undertook data collection through the analysis of the published manuscripts. As a result, in some cases, heterogeneity with regard to the type and specificity of data reported limited the amount of data specific to our cohort of interest that could be extracted and contributed to data loss. Lastly, the present systematic review was limited to published manuscripts in the English language. As such, evidence, including ongoing trials, pre-prints or other grey literature, or those not available in the English language, have not been included in this review.

Despite these limitations, the produced work demonstrates several key strengths. First, present systematic review represents the most recent summary of the literature regarding the use of neuroendoscopy in the setting of metastatic brain lesions, providing an update as to the recent evidence in this space. Second, the authors utilised a broad search strategy and inclusion criteria, which allowed the inclusion and assessment of all relevant studies regardless of study design or size. Lastly, the present study assessed the existing evidence across numerous domains, including participant demographics, procedural factors and outcome measures, including procedural success and adverse events, which are relevant to clinical decision-making and service planning.

Following the completion of the present study, the following future directions for research in this field are proposed arising from limitations of this work. First, it is recommended that future work should stratify reported data by disease histology and procedures undertaken to allow assessment of differential treatment efficacy across these different patient cohorts. Second, it is recommended that the performance of more prospective studies with greater numbers of participants is required in order to increase the generalizability of the study findings and provide a stronger evidence base to inform clinical practice. Third, it is recommended that future work should incorporate greater comparisons and incorporate alternative methods, such as microsurgery, to allow comparisons of efficacy and inform clinical decision-making.

In conclusion, the analysis of the aggregated data revealed that neuroendoscopy provides a similar resection rate and overall survival to microsurgical techniques. However, the strength of the analysis is limited by the quality of the included studies, the majority of which were small in scale and retrospective in nature, and data loss arising from heterogeneity in reported data. From the findings of the present study, several priorities have been identified for future research, including the stratification of outcome measure reporting by disease histology, the performance of large-scale prospective studies, and comparisons to alternative diagnostic and treatment modalities. Overall, the present study demonstrates the value of neuroendoscopy as a tool to facilitate the diagnosis and treatment of metastatic brain lesions, and we look forward to further research in this space.

## Supplementary Material

Patient demographics.

Summary of the procedures performed in the included studies.

Summary of outcomes of procedures.

## Figures and Tables

**Table I:** List of the studies included in the present systematic review.

First author, year of publication	Title of study	(Refs.)
Andreev, 2020	Breast cancer metastasis into a giant hormone-inactive pituitary adenoma (clinical case and literature review).	(7)
Ansari, 2020	The Supraorbital Eyebrow Craniotomy for Intra- and Extra-Axial Brain Tumors: A Single-Center Series and Technique Modification.	(8)
Barkhoudarian, 2017	Microsurgical Endoscope-Assisted Gravity-Aided Transfalcine Approach for Contralateral Metastatic Deep Medial Cortical Tumors.	(9)
Bettag, 2022	Endoscope-assisted visualization of 5-aminolevulinic acid fluorescence in surgery for brain metastases.	(10)
Cathel, 2019	Metastatic Disease to Clivus: Biopsy or Not?	(11)
Ceylan, 2009	Extended endoscopic approaches for midline skull-base lesions.	(12)
Choo, 2018	Neuroendoscopic Cylinder Surgery and 5-Aminolevulinic Acid Photodynamic Diagnosis of Deep-Seated Intracranial Lesions.	(13)
Gazzeri, 2014	Endoscopic supraorbital eyebrow approach for the surgical treatment of extraaxialand intraaxial tumors.	(14)
Hanada, 2010	Metastatic pineal tumors treated by neuroendoscopic surgery-two case reports.	(15)
Hong, 2016	Comparison of endoscope- vs. microscope-assisted resection of deep-seated intracranial lesions using a minimally invasive port retractor system.	(16)
Hu, 2020	Pituitary Metastasis of Pulmonary Large Cell Neuroendocrine Carcinoma: A Case Report.	(17)
Iacoangeli, 2012	Endoscopy-verified occult subependymal dissemination of glioblastoma and brain metastasis undetected by MRI: prognostic significance.	(18)
Jeon, 2018	Endoscopic transorbital surgery for Meckel's cave and middle cranial fossa tumors: surgical technique and early results.	(19)
Jimenez-Vazquez, 2017	Neuroendoscopic and histopathological correlation in 13 cases of cystic fluid filled brain tumours.	(20)
Kassam, 2009	Completely endoscopic resection of intraparenchymal brain tumors.	(21)
Kruljac, 2010	Hypopituitarism caused by pituitary metastasis of supraglottic laryngeal carcinoma: case report.	(22)
Kutlay, 2016	Fully Endoscopic Resection of Intra-Axial Brain Lesions Using Neuronavigated Pediatric Anoscope.	(23)
Kutlay, 2021	Fluorescein Sodium-Guided Neuroendoscopic Resection of Deep-Seated Malignant Brain Tumors: Preliminary Results of 18 Patients.	(24)
Kutlay, 2021	Resection of intra- and paraventricular malignant brain tumors using fluorescein sodium-guided neuroendoscopic transtubular approach.	(25)
Ma, 2018	Endoscopy in Temporal Lobe Glioma and Metastasis Resection: Is There a Role?	(26)
Maeshima, 2022	Hemorrhagic brain metastasis from small-cell carcinoma of the urinary bladder.	(27)
McLaughlin, 2012	Side-Cutting Aspiration Device for Endoscopic and Microscopic Tumor Removal.	(28)
Mitsumasa, 2020	Diplopia Presenting in a Case of Pineal Metastasis of Pulmonary Sarcomatoid Carcinoma Refractory to Treatment.	(29)
Nemoto, 2013	Isolated pineal region metastasis from lung adenocarcinoma with obstructive hydrocephalus: a case report.	(30)
Newman, 2019	Stereotactic-Guided Dilatable Endoscopic Port Surgery for Deep-Seated Brain Tumors: Technical Report with Comparative Case Series Analysis.	(31)
Plaha, 2014	Minimally invasive endoscopic resection of intraparenchymal brain tumors.	(32)
Serra, 2020	Microneurosurgical removal of thalamic lesions: surgical results and considerations from a large, single-surgeon consecutive series.	(33)
Shirane, 2001	Surgical treatment of posterior fossa tumors via the occipital transtentorial approach: evaluation of operative safety and results in 14 patients with anterosuperior cerebellar tumors.	(34)
Souweidane, 2000	Endoscopic Biopsy for Tumors of the Third Ventricle.	(35)
Stamates, 2018	Combined Open and Endoscopic Endonasal Skull Base Resection of a Rare Endometrial Carcinoma Metastasis.	(36)
Villanueva, 2015	Endoscopic and Gravity-Assisted Resection of Medial Temporo-occipital Lesions Through a Supracerebellar Transtentorial Approach: Technical Notes With Case Illustrations.	(37)
Zacharia, 2015	Endoscopic Endonasal Management of Metastatic Lesions of the Anterior Skull Base: Case Series and Literature Review.	(38)
Zagzoog, 2017	Metastatic Liposarcoma of the Skull Base: A Case Report and Review of Literature.	(39)
Zhang, 2018	Clival metastasis of renal clear cell carcinoma: Case report and literature review.	(40)

## Data Availability

The data generated in the present study may be requested from the corresponding author.
