# Supplementary material for: Use of neuroendoscopy in metastatic brain tumours: A systematic review
Source: Med Int (Lond). 2026 Apr 14;6(3):31. doi: 10.3892/mi.2026.315 (PMC13111978; doi:10.3892/mi.2026.315)
Supplement: Patient demographics. [file Supplementary_Data.pdf]

Table SI. Patient demographics.

A, Demographics of the studies included in the present systematic review									
First author, year of publication	Included patients[a]	Total no. of patients	Age, years	Sex (F/M)	Presenting symptoms	Primary cancer/histology	Lesion location	Lesion size	(Refs.)[b]
Andreev, 2020	1	1	55	n/a	1 Acute right-sided ptosis, visual impairment, amnesia, euphoric mood, ataxia	Breast	Endo-supra-retrosellar	n/a	(7)
Ansari, 2020	17	117	n/a	n/a	n/a	n/a	n/a	n/a	(8)
Barkhoudarian, 2017	11	11	60.5 (45-75)	6/5	(7/11) hemiparesis<br>(3/11) asymptomatic<br>(1/11) generalized seizure	(3/11) lung carcinoma<br>(4/11) Melanoma<br>(2/11) Bladder<br>(1/11) Breast<br>(1/11) Ovarian	Deep medial cortical tumours<br><br>(4/11) medial frontal<br>(2/11) precuneus<br>(4/11) paracentral lobule<br>(1/11) cuneus	5.5 cm3 (0.4-16.4)	(9)
Bettag, 2022	26	26	n/a	10/16	n/a	9/26 NSCLC<br>3/26 SCLC<br>3/26 GIT<br>2/26 Breast<br>7/26 Melanoma<br>1/26 Renal<br>1/26 Unknown	16/26 Supratentorial<br>10/26 infratentorial	Max diameter: 33.42±15.32 mm	(10)

Cathel, 2019	1	1	65	0/1	1 right-sided headache and complete ophthalmoplegia of the right eye	Hepatocellular carcinoma	Clivus with invasion into the right cavernous sinus	n/a	(11)
Ceylan, 2009	1	13	53	1/0	n/a	n/a	Midline skull base	n/a	(12)
Choo, 2018	2	20	47.5 (45-50)	2/0	n/a	n/a	1 Temporal<br>1 Cerebellum	1/2 0.74 cm3<br>1/2 6.94 cm3	(13)
Gazzeri, 2014	4	97	n/a	n/a	n/a	n/a	n/a	n/a	(14)
Hanada, 2010	2	2	53 (37-69)	0/2	1 gait disturbance, dementia, and urinary incontinence<br><br>1 vomiting, headache, and consciousness disturbance	1 SCLC<br><br>1 Thyroid	(2/2) Pineal region	23 mm<br>28 mm	(15)
Hong, 2016	2	20	56 (37-75)	1/1	Ataxia	1 Lung<br>1 Breast	(2/2) Rt Cerebellum	n/a	(16)
Hu, 2020	1	1	55	1/0	1 fatigue, dizziness, unsteady balance, bilateral temporal headaches, memory loss and mild mood alterations	Lung Large cell neuroendocrine carcinoma (LCNEC)	Sellar and Suprasellar	10 mm	(17)
Iacoangeli, 2012	1	2	61	1/0	unsteady gait, headache, nausea	GIT	choroid plexus lateral ventricle	n/a	(18)
Jeon, 2018	1	9	54	0/1	n/a	Renal cell carcinoma	Middle cranial fossa	n/a	(19)
Jiminez, 2017	2	13	n/a	2/0	n/a	2/2 Breast	n/a	n/a	(20)
Kassam, 2009	12	21	63 (44-	5/7	n/a	(6/12) NSCLC	(6/12) cerebellum	37.8 mm	(21)

			79)			(3/12) Breast<br>(1/12) Oesophagus<br>(1/12) Renal<br>(1/12) vaginal adenocarcinoma	(1/12) temporal<br>(2/12) parietooccipital<br>(1/12) frontoparietal<br>(1/12) frontal operculum, perisylvian<br>(1/12) parietal		
Kruljac, 2010	1	1	70	0/1	1 Diplopia, syncope, headache, general malaise, loss of appetite	Laryngeal Squamous Cell Carcinoma	Sellar and Suprasellar	35x25x25 mm	(22)
Kutlay, 2016	2	13	47 (44-50)	0/2	Headache	n/a	1 Rt Frontal<br>1 Rt Parietal	2/2 41 mm	(23)
Kutlay, 2021	7	18	57.14 (37-75)	3/4	n/a	4 Lung Adenocarcinoma<br>2 SCLC<br>1 Breast	1 L temporo-parietal<br>1 L frontal<br>1 R parietal<br>1 R caudate nucleus<br>1 R atrium<br>1 L temporal<br>1 L occipital	n/a	(24)
Kutlay, 2021	7	20	59.86 (37-75)	3/4	n/a	5 Lung<br>2 Breast	1 R caudate nucleus<br>1 R atrium<br>2 L atrium<br>2 L temporal horn<br>2 L occipital horn	n/a	(25)
Ma, 2018	11	45	n/a	n/a	n/a	n/a	11/11 Temporal lobe	n/a	(26)
Maeshima, 2022	1	1	76	0/1	1 Aphasia, Hemiplegia	Small cell carcinoma of the urinary	Lt Basal ganglia	n/a	(27)

						bladder			
McLaughlin, 2012	2	31	54.5 (41-68)	1/1	n/a	1 Melanoma<br>1 GIT	n/a	n/a	(28)
Mitsumasa, 2020	1	1	42	1/0	1 Diplopia, headache, nausea, disorientation, urinary incontinence	Lung sarcomatoid carcinoma	Pineal gland	1.9x1.5x1.9 cm	(29)
Nemoto, 2013	1	1	63	1/0	1 Gait disturbance, dementia, urinary incontinence	Lung Adenocarcinoma	Pineal	25 mm	(30)
Newman, 2019	2	16	64.5 (63-66)	0 / 2	n/a	1/2 NSCLC<br>1/2 Esophageal Adenocarcinoma	1/2 Parietal<br>1/2 Basal Ganglia	n/a	(31)
Plaha, 2014	12	48	n/a	n/a	n/a	(10/12) adenocarcinoma (breast, lung, gastrointestinal)<br>(2/12) Melanoma	n/a	n/a	(32)
Serra, 2020	1	92	n/a	n/a	n/a	n/a	Thalamic	n/a	(33)
Shirane, 2001	2	14	65 (60-70)	1/1	1 difficulty walking and headaches	1 Lung	1 left posterior fossa<br>1 Anterosuperior Cerebellum	(1/2) 2.5x2 cm<br>(1/2) n/a	(34)
Souweidane, 2000	1	12	56	n/a	n/a	1 Melanoma	n/a	30 mm	(35)
Stamates, 2018	1	1	60	1/0	1 Headache, Rt Cheek Pain and Numbness	Endometrial carcinoma	Infratemporal fossa, Middle fossa, Cavernous sinus, Trigeminal nerve, and Nasal sinuses	n/a	(36)
Villanueva, 2015	2	4	52 (39-	2/0	1 scintillating scotoma in RT	1 Melanoma	1 Lt Medial Occipital	n/a	(37)

			65)		visual field<br>1 incidental PET finding	1 Breast	Lobe<br>1 Lt Intraventricular extending into posterior medial temporal lobe		
Zacharia, 2015	12	12	64.3 (49-80)	8/4	(6/12) visual loss<br>(5/12) Headache/neck pain<br>(3/12) cranial nerve palsy<br>(1/12) nausea and dizziness<br>(1/12) endocrinopathy	(4/12) Breast<br>(3/12) lung adenocarcinoma<br>(2/12) Thyroid<br>(1/12) bladder adenocarcinoma<br>(1/12) Renal<br>(1/12 prostate carcinoma	(3/12) sellar/suprasellar<br>(3/12) clivus<br>(2/12) sphenoid/ethmoid sinuses<br>(1/12) suprasellar<br>(1/12) cavernous sinus<br>(1/12) craniovertebral junction<br>(1/12) sellar	n/a	(38)
Zagzoog, 2017	1	1	43	1/0	1 horizontal diplopia with right lateral gaze, complete right sixth nerve palsy	Lt Scapular myxoid liposarcoma	Sellar and parasellar	3.9x2.8x3.7 cm	(39)
Zhang, 2018	1	1	54	0/1	asthenia, ptosis, diplopia	Renal cell carcinoma	clivus	n/a	(40)

B, Summary of the data in the included studies

	Included patients[a]	Total no. of patients	Age, years	Sex (F/M)	Presenting symptoms	Primary cancer/histology	Lesion location	Lesion size	
	Total: 150<br><br>Average: 4.41	Total: 686<br><br>Average:	Average: 57.46 (37-80)	51/54<br><br>Female:	Reported (18/34)<br><br>Presenting symptoms varied	Reported 27/34<br><br>42/113 (37.17%) Lung	**Reported (29/34)**<br><br>**Supratentorial - (93/115)**	Reported (13/34)	

	(1-26)<br><br>15 of the included papers only had 1 patient who has undergone neuroendoscopy for the biopsy or resection of a metastatic brain tumour	20.17 (1-17)<br><br>150/686 (21.87%) of patients in the included studies have undergone neuro-endoscopy for the biopsy or resection of a metastatic brain tumour		51/105 (48.57%)<br><br>Male: 54/105 (51.43%)		- 26/113 (23.01%) NSCLC<br>- 6/113 (5.31%) SCLC<br>- 10/113 (8.75%) Unclassified<br>18/113 (15.93%) Breast<br>16/113 (14.16%) Melanoma<br>10/113 (8.75%) Unclassified Adenocarcinoma (Breast, Lung, GIT)<br><br>**OTHER 27/113 (23.89) (ALL BELOW THIS LINE)**<br>5/113 (4.42%) Renal<br>- 3/113 (2.65%) Unclassified<br>- 2/113 (1.77%) RCC<br>5/113 (4.42%) GIT<br>4/113 (3.54%) Bladder<br>- 2/113 (1.77%) Unclassified<br>- 1/113 (0.88%) Small Cell<br>- 1/113 (0.88%) Adenocarcinoma<br>3/113 (2.65%) Thyroid<br>2/113 (1.77%) Oesophagus<br>- 1/113 (0.88%) Adenocarcinoma<br>- 1/113 (0.88%) Unclassified<br>1/113 (0.88%) Prostate	**Ventricular (10/115)**<br><br>1 R atrium (lat ventricular)<br>1 R atrium (lat ventricular)<br>2 L atrium (lat ventricular)<br>2 L temporal horn (lat ventricular)<br>2 L occipital horn (lat ventricular)<br>1 Lt Intraventricular extending into posterior medial temporal lobe<br>1 choroid plexus lateral ventricle<br><br>**Cortical (38/115)**<br><br>(1/12) temporal<br>(2/12) parietooccipital<br>(1/12) frontoparietal<br>(1/12) frontal operculum, perisylvian<br>(1/12) parietal<br>(4/11) medial frontal	Max dimension (8/13) - Range: 10-41mm<br><br>Dimensions (4/13)<br>Volume (1/13)	

						1/113 (0.88%) Vaginal Adenocarcinoma<br>1/113 (0.88%) Ovarian<br><br>1/113 (0.88%) Endometrial<br>1/113 (0.88%) Laryngeal SCC<br>1/113 (0.88%) Scapular Myxoid Liposarcoma<br>1/113 (0.88%) Hepatocellular Carcinoma<br><br>1/113 (0.88%) Unknown	(2/11) precuneus<br>(4/11) paracentral lobule<br>(1/11) cuneus<br>1 L temporo-parietal<br>1 L frontal<br>1 R parietal<br>1 L temporal<br>1 L occipital<br>1 Rt Frontal<br>1 Rt Parietal<br>1 Temporal<br>1 Lt Medial Occipital Lobe<br>1/2 Parietal<br>11/11 Temporal lobe<br><br>**Subcortical (9/115)**<br><br>1 R caudate nucleus<br>1 R caudate nucleus<br>2 Pineal Region<br>1 Lt Basal Ganglia<br>1 Pineal<br>1 Pineal Gland<br>1 Thalamic		

							1/2 Basal Ganglia<br><br>**Sinuses (4/115)**<br><br>(1/12) intraorbital extension /infratemporal fossa /ethmoid and sphenoid sinuses<br>(1/12) ethmoid and sphenoid sinus/lamina papyracea/intracranial<br>(1/12) cavernous sinus<br>1 Infratemporal fossa, Middle fossa, Cavernous sinus, Trigeminal nerve, and Nasal sinuses<br><br>**Sellar (14/115)**<br>(1/12) suprasellar<br>(1/12) sellar)<br>(3/12) sellar/suprasellar<br>(3/12) clivus)<br>1 Sellar and Suprasellar<br>1 Sellar and Parasellar<br>1 Sellar and Suprasellar		

							1 Endo-supra-retrosellar<br>1 Clivus with invasion into the right cavernous sinus<br>1 clivus<br><br>**Other (18/115)**<br>1 Middle Cranial Fossa<br>16/26 Supratentorial<br>1 Midline Skull Base<br><br>**Infratentorial (22/115)**<br><br>**Cerebellum (10/115)**<br>1 Anterosuperior Cerebellum<br>(6/12) cerebellum<br>2 Rt Cerebellum<br>1 Cerebellum<br><br>**Other (12/115)**<br>1 left posterior fossa<br>(1/12) craniocervical junction<br>10/26 infratentorial		

[a]Included patients refers to those patients who have undergone neuroendoscopy for the biopsy or resection of a metastatic brain tumour, and are therefore included in the analysis. The total no. of patients refers to the total number of patients included in the study regardless of the diagnosis or procedure undergone. [b]Reference citations refer to the reference list in the main manuscript.

Table SII. Summary of the procedures performed in the included studies.

A, Procedures in the included studies										
First author, year of publication	Biopsy vs. resection	Combined with open vs. fully endoscopic	Rigid vs. flexible endoscope	Route and technique	Fluorescence	Adjuvant chemotherapy	Adjuvant radiotherapy	Preoperative assessment	Post-operative assessment	(Refs.)[a]
Andreev, 2020	Both	Fully endoscopic	n/a	Extended endonasal	n/a	n/a	n/a	MRI	CT on 1st postoperative day<br>MRI on 11th postoperative day	(7)
Ansari, 2020	Both	Combined	Rigid	Transorbital	Y	n/a	n/a	MRI	MRI (immediate and 3 month)	(8)
Barkhoudarian, 2017	Resection	Combined	n/a	Transcranial	N	n/a	n/a	MRI (with contrast enhanced)	MRI within 24-48 h<br>Neurological assessment	(9)
Bettag, 2022	Both	Combined	n/a	Transcranial	Y	n/a	n/a	MRI (T1 weighted)	n/a	(10)
Cathel, 2019	Biopsy	Fully endoscopic	Rigid	Extended endonasal	N	n/a	n/a	MRI	n/a	(11)
Ceylan, 2009	Resection	Fully endoscopic	n/a	Extended endonasal	n/a	n/a	n/a	MRI (1.5-T contrast-enhanced magnetic resonance imaging)	n/a	(12)

Choo, 2018	Resection	Fully endoscopic	Rigid	n/a	Y (5-ALA)	n/a	n/a	MRI (T1 and T2 Weighted)	CT within 24 h to assess for haemorrhages<br><br>Postoperative MRI within 1 week (T1 weighted, non-contrast enhanced and contrast enhanced)	(13)
Gazzeri, 2014	Resection	Fully endoscopic	Rigid	Transorbital	n/a	n/a	n/a	MRI and/or head CT	Contrast-enhanced MRI within 48 h after surgery and 6 weeks post-operatively	(14)
Hanada, 2010	Biopsy and ventri-culostomy	Fully endoscopic	n/a	Transcranial	n/a	n/a	Y	1 CT, MRI, PET<br>1 CT, MRI	MRI	(15)
Hong, 2016	Resection	Fully endoscopic	Rigid	n/a	n/a	n/a	n/a	n/a	MRI within 24 h<br><br>T1-weighted<br>and T2-weighted FLAIR images 2 or<br>more months after surgery	(16)
Hu, 2020	Resection	Fully endoscopic	n/a	Extended endonasal	n/a	N	Y	MRI & CT	MRI	(17)
Iacoangeli, 2012	Resection	Fully endoscopic	Rigid	Transcranial	N	Y	Y	MRI	CT, MRI, PET, gastroscopy, colonoscopy, capsule endoscopy	(18)

Jeon, 2018	Resection	Fully endoscopic	Rigid	Transorbital	n/a	n/a	n/a	MRI and CT	MRI	(19)
Jiminez, 2017	Biopsy	Combined	Rigid	Transcranial	n/a	n/a	n/a	n/a	n/a	(20)
Kassam, 2009	Resection	Fully endoscopic	Rigid	Transcranial	N	n/a	n/a	MRI (contrast enhanced), (CT in one case)	Intraoperative CT (non-cerebellar)<br><br>MRI w/w/out IV contrast<br><br>Neurological assessment	(21)
Kruljac, 2010	Resection	Fully endoscopic	n/a	Extended endonasal	n/a	N	Y	MRI	n/a	(22)
Kutlay, 2016	Resection	Fully endoscopic	Rigid	Transcranial	n/a	n/a	n/a	T1 Weighted MRI	Postoperative CT on first post-operative day<br><br>MRI with and without IV contrast within 2 days after surgery	(23)
Kutlay, 2021	Resection	Fully endoscopic	Rigid	Transcranial	Y (Fluorescein sodium guided)	n/a	n/a	KPS Score<br>MRI	KPS Score at discharge and 3M follow-up<br><br>MRI within 48 hours after surgery	(24)
Kutlay, 2021	Resection	Fully endoscopic	Rigid	Transcranial	Y (Fluorescein sodium	n/a	n/a	KPS Score<br>MRI	KPS Score at discharge and 3M follow-up	(25)

					guided)				MRI within first 24-48 h after surgery	
Ma, 2018	Resection	Fully endoscopic	Rigid	Transcranial	Y (0/11)	N	N	MRI (volumetric T1-weighted, DTI, T2 weighted, fluorescence *in situ* hybridization (FLAIR)	MRI within 24-48 h	(26)
Maeshima, 2022	Resection	Fully endoscopic	Flexible	Transcranial	n/a	N	Y	CT & MRI (T1 and T2 Weighted, Gadolinium Enhanced)		(27)
McLaughlin, 2012	Resection	Fully endoscopic	Rigid	Transcranial	n/a	n/a	n/a	T1 Weighted post gadolinium MRI	T1 Weighted post gadolinium MRI	(28)
Mitsumasa, 2020	Biopsy and ventri-culostomy	Fully endoscopic	Flexible	Transcranial	n/a	n/a	n/a	MRI (contrast)	n/a	(29)
Nemoto, 2013	Biopsy and ventri-culostomy	Fully endoscopic	Flexible	Transcranial	N	Y	Y	CT & MRI (T1 & T2 Weighted)	PET CT<br>MRI	(30)
Newman, 2019	Resection	Combined	n/a	Transcranial	n/a	n/a	n/a	MRI<br>CT	Immediate CT<br>Delayed MRI at 3 months	(31)

Plaha, 2014	Resection	Fully endoscopic	Rigid	Transcranial	N	n/a	n/a	MRI (T1-weighted contrast-enhanced)	CT, MRI, or FLAIR MRI	(32)
Serra, 2020	Resection	Combined	n/a	Transcranial	Y (not helpful)	n/a	n/a	Complete neurological examination<br><br>3-T MRI (Tractography)	Complete Neurological Examination at discharge and 3 months<br><br>3-T MRI (Tractography) at 3 months	(33)
Shirane, 2001	Resection	Fully endoscopic	n/a	Transcranial	n/a	n/a	Y (1/2)	MRI (Gadolinium-enhanced T1 weighted)	MRI (Gadolinium-enhanced T1 weighted)<br><br>CT	(34)
Souweidane, 2000	Biopsy	Fully endoscopic	Rigid	Transcranial	n/a	n/a	n/a	MRI with gadolinium	MRI or CT within 48 hours of procedure	(35)
Stamates, 2018	Resection	Combined	n/a	Transorbital and extended endonasal	n/a	Y	Y	MRI	MRI	(36)
Villanueva, 2015	Resection	Combined	n/a	Transcranial	n/a	Y (1/2)	N	MRI	MRI	(37)
Zacharia, 2015	Both	Fully endoscopic	Rigid	Extended endonasal	Y (to detect CSF leaks)	Y (11/12)	Y (11/12)	MRI (Gadolinium enhanced)	MRI (Gadolinium enhanced)	(38)
Zagzoog, 2017	Resection	Fully	n/a	Extended	n/a	n/a	Y	CT and MRI (T1	MRI at 3 months	(39)

		endoscopic		endonasal				Weighted, Gadolinium Enhanced)		
Zhang, 2018	Both	Fully endoscopic	Rigid	Extended endonasal	N	N	Y	MRI, CT	CT	(40)
B, Summary of information from the included studies										
	Biopsy vs. resection	Combined with open vs. fully endoscopic	Rigid vs. flexible endoscope	Route and technique	Fluorescence	Adjuvant chemotherapy	Adjuvant radiotherapy	Preoperative assessment	Post-operative assessment	
	Of 34 studies included, the goal of procedure was: 23/34 Resection (67.65%) 5/34 Both Resection	34/34 reported 26/34 (76.47%) Fully Endoscopic 8/34 (23.53%) Combined	18/21 (85.71%) Rigid 3/21 (14.29%) Flexible	32/34 reported 20/32 Transcranial 8/32 Extended endonasal 3/32 Transorbital 1/32 transorbital and extended	7/15 Y 8/15 N 15/34 reported	5/10 Y 5/10 N 10/34 reported	11/ 13 Y 2/13 N 13/34 reported	32/32 MRI 9/32 CT 32/34 reported	26/27 MRI 11/34 CT 27/34 reported	

	and Biopsy (14.71%)<br><br>3/34 Biopsy (8.82%)<br><br>3/34 Biopsy and ventri-culostomy (8.82%)			endonasal						

[a]Reference citations refer to the reference list in the main manuscript.

Table SIII. Summary of outcomes of procedures.

A, List of included studies and outcomes							
First author, year of publication	Procedure success	Duration of surgery	Adverse events	Hospital stay	Follow-up	Clinical outcomes	(Refs.)[a]
Andreev, 2020	STR	n/a	n/a	14 days	14 days	Amaurosis of the right eye<br>Certain regression of psycho-emotional and amnestic disorders	(7)
Ansari, 2020	n/a	n/a	1/17 multiorgan failure death<br>1/17 PE death	n/a	n/a	n/a	(8)
Barkhoudarian, 2017	8/11 GTR<br>3/11 NTR	n/a	None	n/a	n/a	6/11 Improved symptoms	(9)
Bettag, 2022	Endoscope visible fluorescence: 22/26 (21/22 true positive)	n/a	2/26 wound infection<br>2/26 transient arm paresis	n/a	11.33±8.14 months (2-17)	Decrease<br><br>Mean preoperative KPS: 91.9% ± 9.8% a<br>Mean discharge KPS: 81.2% ± 23.4% (p = 0.006)<br><br>Mean overall survival: 11.04±8.65 months	(10)

Cathel, 2019	Biopsy obtained	n/a	n/a	n/a	n/a	n/a	(11)
Ceylan, 2009	GTR	n/a	n/a	n/a	29 months	n/a	(12)
Choo, 2018	2/2 GTR	n/a	n/a	n/a	n/a	n/a	(13)
Gazzeri, 2014	n/a	n/a	n/a	n/a	n/a	n/a	(14)
Hanada, 2010	Biopsy obtained and hydrocephalus resolved	n/a	None	n/a	6 months	(2/2) Improved	(15)
Hong, 2016	1/2 GTR<br>1/2 NTR	n/a	n/a	n/a	n/a	n/a	(16)
Hu, 2020	STR	n/a	None	n/a	12 months	Improved symptoms<br>Signs and symptoms of diabetes insipidus (Polyuria, Hypernatremia, Low urine Specific Gravity) responding to DDAVP	(17)
Iacoangeli, 2012	n/a	n/a	n/a	n/a	n/a	Overall survival: 20 months	(18)
Jeon, 2018	GTR	n/a	n/a	n/a	n/a	Improved	(19)
Jiminez, 2017	n/a	n/a	n/a	n/a	n/a	n/a	(20)
Kassam, 2009	4/12 GTR<br>4/12 NTR<br>4/12 STR	n/a	1/12 brain abscess + pulmonary embolism<br>1/12 re-cannulation (missed tumour first time)	n/a	Median 5 months (2-24)	n/a	(21)

Kruljac, 2010	STR	n/a	n/a	n/a	n/a	n/a	(22)
Kutlay, 2016	2/2 GTR	n/a	n/a	n/a	n/a	n/a	(23)
Kutlay, 2021	6/7 GTR<br>1/7 NTR	124.43 min (85-160)	None	n/a	3 months	n/a	(24)
Kutlay, 2021	6/7 GTR<br>1/7 NTR	119.29 min (83-160)	None	n/a	3 months	n/a	(25)
Ma, 2018	7/11 GTR<br>2/11 NTR<br>2/11 STR	n/a	1/11 CSF leak	n/a	n/a	11/11 Median overall survival 12.9 months<br><br>11/11 Improved	(26)
Maeshima, 2022	STR	n/a	n/a	n/a	4 months	Aphasia & Rt Hemiplegia not improved<br><br>Condition gradually deteriorated and died 4 months postoperative	(27)
McLaughlin, 2012	2/2 GTR	n/a	n/a	n/a	n/a	n/a	(28)
Mitsumasa, 2020	Biopsy obtained	n/a	n/a	n/a	n/a	n/a	(29)
Nemoto, 2013	Biopsy obtained and hydrocephalus resolved	n/a	n/a	n/a	3 months	Gradual improvement of the hydrocephalus, the patient was able to communicate and walk with assistance	(30)
Newman, 2019	2/2 GTR	n/a	None	n/a	3 months	n/a	(31)

Plaha, 2014	11/12 GTR<br>1/12 STR	n/a	1/12 wound infection	n/a	n/a	n/a	(32)
Serra, 2020	n/a	n/a	n/a	n/a	3 months	n/a	(33)
Shirane, 2001	2/2 GTR	n/a	n/a	n/a	n/a	(2/2) Improved - excellent postoperative outcomes	(34)
Souweidane, 2000	n/a	n/a	n/a	n/a	n/a	n/a	(35)
Stamates, 2018	STR	n/a	n/a	n/a	12 months	Improved - Alive and Well at 12 months	(36)
Villanueva, 2015	1/2 GTR<br>1/2 NTR	n/a	1/2 Proximal optic radiation infarct	n/a	(1/2) 30 months	(1/2) improved	(37)
Zacharia, 2015	4/10 GTR<br>6/10 STR<br>2/2 Biopsy	n/a	7/12 intraoperative CSF leak<br><br>3/12 panhypopituitarism	n/a	14 months (1-55)	Median overall survival: 16 months<br><br>Median progression free survival: 18 months	(38)
Zagzoog, 2017	STR	n/a	None	n/a	3 months	Minimal movement of the right eye outwards, diplopia improved marginally	(39)
Zhang, 2018	Biopsy (1/1)<br>STR (1/1)	n/a	1/1 bleed requiring 400 ml transfusion	n/a	4 months	Small improvement in symptoms	(40)

B, Summary of outcomes							
	Procedure success	Duration of surgery	Adverse events	Hospital stay	Follow-up	Clinical outcomes	
	Of studies reporting resection as goal:<br><br>23/28 report GTR/NTR/STR<br><br>60/93 GTR (64.51%)<br>13/93 NTR (13.98%)<br>20/93 STR (21.50%)<br><br>Of studies reporting biopsy as goal:<br><br>6/6 report success of biopsy<br><br>7/7 Biopsy Obtained (100%)<br><br>6/34 report no outcome measures	Only reported in 2 studies by same group:<br><br>121.86 mins (83 - 160)	In studies where complications are reported, across 124 patients, adverse events reported include:<br><br>**Intraoperative Complications:**<br>7 Intraoperative CSF leaks<br>1 intraoperative hemorrhage requiring 400mL transfusion<br><br>**Post-operative Complications:**<br>3 wound infections<br>3 panhypopituitarism<br>2 Transient arm paresis<br>1 Postoperative CSF leak<br>1 brain abscess + pulmonary embolism<br>1 re-cannulation (missed tumour first time)<br>1 Proximal optic radiation infarct<br>1 multiorgan failure, death<br>1 PE death<br>.	Only reported in 1 study:<br><br>14 days	Wide range depending on study criteria and case outcomes after surgery:<br><br>14 days - 55 months	Symptoms<br><br>11 studies report symptomatic improvement<br>2 report no improvement/decline<br><br>28/34 (82.35%) of patients were reported to have at least some level of symptomatic improvement.<br><br>Average of overall survival reported in 4 studies (50 patients) is 14.99 months. When weighted by the number of patients in each study, the average overall survival is 12.82 months.	

[a]Reference citations refer to the reference list in the main manuscript. GTR, gross total resection; NTR, near total resection; STR, subtotal resection.	
